# Possible Effects on Health of Ultrasound Exposure, Risk Factors in the Work Environment and Occupational Safety Review

**DOI:** 10.3390/healthcare10030423

**Published:** 2022-02-24

**Authors:** David Baeza Moyano, Daniel Arranz Paraiso, Roberto Alonso González-Lezcano

**Affiliations:** 1 Department of Chemistry and Biochemistry, Campus Montepríncipe University San Pablo CEU, Alcorcón, 28668 Madrid, Spain; baezams@ceu.es; 2 Department Pharmaceutical and Health Sciences, Knowledge Area Pharmaceutics and Pharmaceutical Technology, Campus Montepríncipe, University San Pablo CEU, Alcorcón, 28668 Madrid, Spain; daniel.arranzparaiso@ceu.es; 3 Architecture and Design Department, Escuela Politécnica Superior, Campus Montepríncipe, University San Pablo CEU, Alcorcón, 28668 Madrid, Spain

**Keywords:** occupational safety, indoor environmental quality, environmental health, occupational hazard, occupational ultrasound, noise exposure, workplace exposure

## Abstract

Ultrasonic waves are mechanical waves with a frequency greater than 20,000 Hz. Ultrasonic waves are emitted by devices that are used in industry or that have a medical or aesthetic purpose. There is growing interest in the effect of ultrasound absorption on the human body, since people’s exposure to these acoustic waves has increased considerably in recent years. There are more and more devices that emit ultrasounds used for different sanitary procedures, aesthetic treatments and industrial processes, creating more possibilities of ultrasound noise, and therefore an increased risk of occupational hazard and occupational danger. Experiments on animals have shown damage to internal organs from receiving different ultrasonic frequencies. The main task of this work was to organize and summarize recent studies on ultrasound to reflect the current state of this technique and establish a systematic basis for future lines of research. This work has allowed us to better understand the unknown field of these high frequencies of sound, and highlights the need to carry out more studies on the ultrasound emissions that can be absorbed by the human body to determine how this energy could affect humans by calculating the maximum dose of exposure and developing manuals for the use of ultrasound-emitting equipment to protect the health of workers and all people. It is necessary to develop regulations by public administrations to improve the protection of workers, health professionals, patients and all people in general for better occupational safety, indoor environmental quality and environmental health.

## 1. Introduction

Lazzaro Spallanzani (Italy) demonstrated that bats are blind animals who navigate in the dark using inaudible sounds (the beginning of echolocation as a concept); because of this discovery, Spallanzani is considered the “father of ultrasound”, even though his theory was highly criticized because during his life, the only known acoustic waves were audible and bat flight was silent [1].

The human ear can detect sounds in the frequency range of 20–20,000 Hz. Sounds that are emitted over this range and are not perceived by the human ear are known as ultrasound. This type of sound can produce a series of harmonic and subharmonic frequencies within the hearing range. Thus, studies on the effects of ultrasound on health are usually performed not only in isolation but also using high- and very high-frequency sounds (generally from 10 kHz), alongside ultrasound in a stricter physical sense (with a frequency greater than 20 kHz).

Ultrasound, as a wave movement, has essentially the same physical properties as sound waves. However, due to their higher frequency and shorter wavelengths, ultrasound waves diffract less than audible sound waves and become more rapidly absorbed by the air; consequently, ultrasound waves are not transmitted over very long distances.

The velocity of the propagation of ultrasound in the air at room temperature is equal to that of audible sounds, with a value around 343 m/s, while their velocity of propagation in a liquid medium such as water is greater, with a value of approximately 1500 m/s. All these properties have the same importance during evaluations as they have during exposure from labor (INSHT, 1984) [2].

Ultrasound waves, when propagating through a medium, transmit energy via the shock between particles. Part of this energy is lost or redistributed through various mechanisms such as absorption, dispersion, loss of viscosity or thermal conduction [3,4,5].

An ultrasound is defined as a series of mechanical waves, generally longitudinal, originating from the vibrations of an elastic body and propagated by a material medium (body tissues), whose frequency exceeds that of the sound that is audible to humans: 20,000 cycles/second or 20 kilohertz (20 kHz). Some of the parameters often used for ultrasound are frequency, propagation speed, interaction of the ultrasound with the tissues and pulse-repetition frequency [4]. 

The human ear is sensitive to frequencies about between 20 Hz and 20 kHz, with a level quantity between 0 dB and 140 dB. The human ear is more sensitive to mid-range frequencies and less sensitive to low and high frequencies [6]. It is normal to maintain these frequency limits for audible sound between 20 Hz and 20 kHz, but these limits will depend on the sensitivity of each person [3].

According to the NTP 205 Ultrasound, Table 1: Occupational Exposure [2] guidelines, the sources of ultrasound generation can be classified as follows according to their frequency: low frequency (10–100 kHz), with many applications from an industrial perspective; medium frequency (100 kHz–1 MHz), for use in therapeutic applications; and high frequency 1–10 MHz), mainly used for medical purposes and nondestructive control devices.

The definition of ultrasound in the occupational and health context is neither uniform nor precise. According to the Health Protection Agency and another international organization, ultrasound is defined as sound above 20 kHz. [6] Although not concretely specified, this limit is the most widely accepted. Ultrasound is emitted into the air by different types of machines [7,8,9]. Some machines emit ultrasound directly (e.g., cutting machines or cleaning baths), and some generate it as a by-product of basic operations (e.g., high-speed drills and pressurized air). Typical working frequencies are 20 kHz, 31.5 kHz, 35 kHz and 40 kHz [9,10]. Table 1 summarizes some of the most common ultrasonic applications, as well as their frequency and intensity ranges.

Because exposure to ultrasound is not explicitly regulated, ultrasound measurements are usually performed when workers complain of discomfort associated with such exposure or when requested by employers who have employees exposed to ultrasound. In Germany, the Social Accident Insurance Institution is usually responsible for carrying out these measures for employers [10,11,12].

Ultrasonic waves are used in many areas, such as hydrolocation and underwater telecommunication, industry and medicine [13]. The frequency range of ultrasound is wide and depends on its use (e.g., from 20 kHz from industrial devices to 10 MHz for medical diagnostics and therapy). The frequency separating the different biological impact mechanisms of ultrasound in the human body is 100 kHz. The latest technological innovations have considerably increased the field of application of USs, so the possible risks that they may present to exposed persons must be considered [13]. 

**Figure 1:**
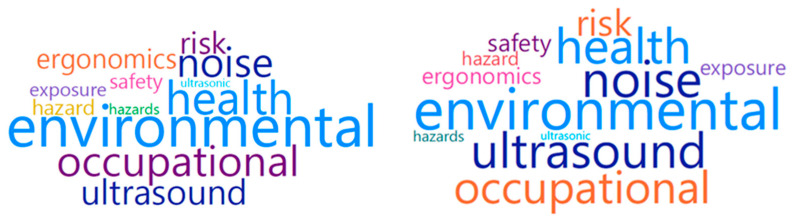
Keywords for issues related to the effect of ultrasound on environmental and occupational health in the period of 1900–2000 (**Left**) and in the last 5 years (**Right**).

Certain workers, because of their professions, are exposed to these nonaudible sound waves in their daily lives. Ultrasonic waves are used in the health sector to obtain images (echography) and for medical and aesthetic treatments. Consequently, it would be interesting to determine the possible effects of ultrasound absorption on humans and what limits people can tolerate before ultrasound becomes harmful to their health. Research and practice in the field of occupational safety has become a relevant issue in the search for environmental health in the workplace, which is affected by various factors, including the effect of ultrasound. Figure 1 shows a keyword search of articles and abstracts related to these areas of research carried out through the Science Web in a period of 100 years and in the last 5 years (Figure 1).

## 2. Materials and Methods

The criteria used for the bibliographic search were homogenized in order to have a common criterion for searching and filtering the information accessed through PubMed, ScienceDirect and Scopus. Keywords and their synonyms or derivatives “ultrasound”, “cavitation”, “bubbles”, “waves”, “hematological”, “genetics” and “guideline” were used as search criteria. These were used in combination with the inclusion criteria “risk factors”, “frequency”, “diagnostic”, “occupational”, “safety”, “animal”, “fetuses”, “temperature”, “legislation”, “propagation”, “effects”, “workplace”.

**Figure 2:**
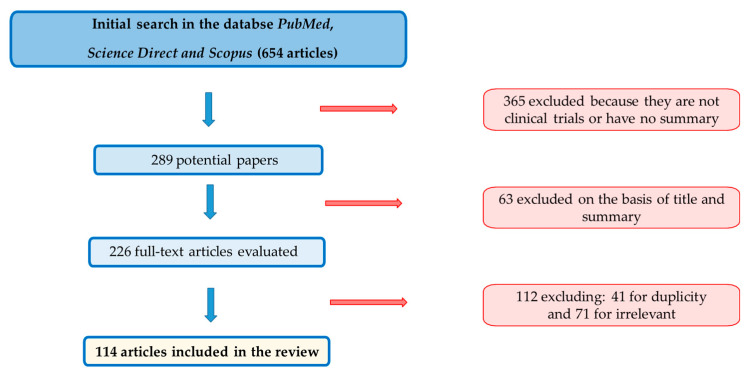
PRISMA flow chart for literature search and selection of articles.

Figure 2 shows the process flow diagram that has been maintained throughout the development of this manuscript.

In terms of the methodology used for the selection of the articles, it was taken into account that the reference articles were no more than five years old from the date of the search (with exceptions such as legislation, guidelines or references to established theories) and were publications dealing with ultrasound and its incidence on living beings, maximum exposure standards for indoor, outdoor, therapeutic and workplace ultrasound exposures, all of which had to be written in English or with an official version published in English, with the exception of laws or guidelines that have been used in the language of the country where they are published.

As for the exclusion criteria considered for the elaboration of the work, publications that did not have the key word “ultrasound” or “exposure” were excluded.

## 3. Results

### 3.1. Propagation and Absorption of Ultrasound

Ultrasound mechanical waves propagate similarly to audible-sound mechanical waves through the displacement of the molecules that make up the medium in which the waves move. Ultrasound waves can propagate in the same direction as particles (longitudinal waves) or transversely or perpendicularly. Longitudinal waves are the most important for ultrasound medical applications [14]; the effects and the mechanisms of their generation are set out in Table 2. Higher-frequency waves are better absorbed than lower-frequency waves. The amplitude and intensity of the wave reduces with distance at a rate of about 0.5 dB cm^−1^ MHz^−1^; for a 3.5 MHz wave, the amplitude will be reduced by one-half, and the intensity by a factor of four (−6 dB) after traveling about 4 cm [14]. 

**Figure 3:**
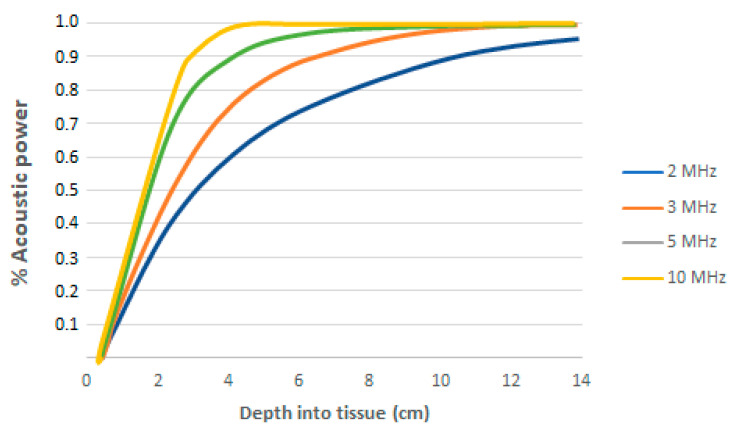
The fraction of the acoustic power leaving a transducer deposited in soft tissue up to a particular depth, depending on frequency. An absorption coefficient of 0.5 dB cm^−1^ MHz^−1^ has been assumed [6].

Figure 3 shows the depth of an ultrasonic wave in cm in the human body as a function of its frequency.

For most diagnostic beams, 90% of the power is deposited within the first 5 cm of tissue. Essentially all the acoustic power entering through the skin surface is absorbed in the body tissues [6]. Speed through tissue depends on fat, collagen and water content [14].

Ultrasounds cause an increase in the speed of biochemical reactions by increasing temperature [16]. For ultrasound propagation through the human body, the lower the acoustic impedance (AI) is, the easier the waves advance. The greater the AI, the greater the reflection. Therefore, coupling gels with an appropriate AI are used. The ultrasound transducer head must always be in motion to avoid temperature increase in the internal place where it is located. 

Table 3 shows the propagation velocities (m s^−1^) of the US through different media and tissues of the human organism [14,17].

The effectiveness in achieving the proposed objective depends on the quality of the apparatus, the phenomena of absorption and reflection and the nature of the tissues it passes through. The concentration of proteins increases the absorption of ultrasound, so tissues with greater collagen content absorb more energy. It is worth considering that in physiotherapy manuals, it is recommended to use the frequency of 3 MHz waves to effectively act up to 4 cm depth, while 1 MHz waves effectively reach up to approximately 12 cm. Some authors use waves between 3 and 5 cm, while others consider those up to 10 cm. The ultrasound scanning technique uses a 50 Hz pulse frequency (noncontinuous) with a 1 MHz carrier frequency to penetrate about 15 cm [18]. 

One of the physical effects or treatments applied in connection with ultrasound is called cavitation, several phenomena that produce the creation, oscillation, growth and shock of bubbles within a medium. When a gas bubble in a liquid experiences the variations in pressure of an acoustic wave, its size is driven to change, expanding during the period of decreased pressure and contracting during the compression half-cycle of the wave. This behavior is termed acoustic cavitation. For low values of peak acoustic pressure, oscillations in bubble radius largely follow variations in pressure. As the peak acoustic pressure increases, the bubble becomes unstable as it contracts, collapsing catastrophically under the inertia of the surrounding liquid. Such cavitation is therefore termed “inertial” to distinguish it from stable or non-inertial cavitation. The adiabatic conditions associated with extremely rapid bubble compression during inertial cavitation result in very high instantaneous temperatures within the bubble. It is highly improbable that either form of cavitation can be generated at diagnostic levels within soft tissues or fluids in the body, in the absence of gas-filled ultrasound contrast agents. However, there are two conditions when the presence of gas may result in mechanical trauma to adjacent soft tissue, caused by a cavitation-like process, at the surface of the lung, and in the intestine [14]. Sound waves can cause bubbles to expand or contract rhythmically. When the bubbles collide, they send out secondary sound waves in all directions. These secondary sound waves can improve the ultrasound image because the wave will reflect to the transducer, giving it more information [19]. 

The physical, chemical and biological effects of cavitation depend on the type of cavitation (inertial, non-inertial, injection or fragmented) and where the ultrasound is applied. The effects also depend on where the bubble is located and its size. When there is high attenuation, the main amplitude of the sound wave increases to obtain a good signal/noise ratio, but this increase does not occur in the detected signal, so the propagation will start to become nonlinear. In this way, the energy will exceed the fundamental frequency as the harmonics increase. These frequencies will be higher than human detection limits (300 kHz), so this energy will become invisible. By increasing the main amplitude of the sound wave, the effect it has on humans increases [20].

Encapsulated microbubbles can break when placed in a moderate ultrasonic field, freeing the bubbles. These bubbles, already free, respond more strongly to ultrasound (close to the size of a bubble), allowing them to enter resonance. The change in the radius of the bubbles is 300% for a 1 MHz ultrasound and 50% for 2 MHz; however, in the encapsulated bubbles, there is only a 3% change [19].

Ultrasounds are also pulsed. The use of high power causes the rapid transformation of one energy into another, which can saturate tissues and cause damage. This phenomenon is detectable by ultrasonic puncture when there is damage to the nerve endings. Consequently, some scholars recommend the application of low power with an increased time of application [21].

Table 4 shows the classification of ultrasound equipment for ultrasound imaging. They have different penetration capacities depending on the plane of the wave jet. They are also used at different frequencies depending on the type.

Members of the independent Advisory Group on Non-Ionizing Radiation [6] stated that ultrasound can have thermal effects, depending on the energy that flows over a long period of time; and mechanical effects, which depend on the amplitude of the pulse.

All ultrasound devices usually come with two measurements [25]:

(1) A Mechanical index (MI), which is the index that indicates the mechanical damage that the device can produce (i.e., from what value the inertial cavitation starts).
(2) Thermal index (TI), whose value, if exceeded, produces damage by heating the tissue.

Both indices consider the properties of the tissue where the ultrasound will be applied and the biophysical process. The methods of measurement are described in the Acoustic Output Measurement Standard for Diagnostic Ultrasound Equipment. The methods of computation are described in the Standard for Real-Time Display of Thermal and Mechanical Acoustic Output Indices on Diagnostic Ultrasound Equipment [26,27].

The Health Protection Agency (HPA) [6] states that high levels of exposure to ultrasound can produce permanent damage to biological tissues. However, at low levels, such as those used in diagnostic testing, they should not produce damage because they do not produce more heat than physiological thermal temperature.

Ultrasound exposure producing a temperature increase greater than or equal to 41 °C for 5 min or longer is potentially dangerous. A limit of intensity levels less than or equal to 137 dB prevents warming of any part of the body [25].

### 3.2. Possible Effects on Humans

The advisors of the independent non-ionizing radiation group observed that the propagation of waves at a frequency higher than 300 kHz through the air is limited to one millimeter due to the absorption of the medium. Thus, the authors established that this ultrasound wave can only have effects on human tissue if it is accompanied by a liquid or solid placed between the device that emits the ultrasound and the body tissue. The heating produced in the tissue will depend on the thickness of the tissue being treated, the thermal conductivity of the tissue and the effect of blood pressure. Calcified bones absorb more ultrasound wave energy, so the adjacent tissue will be warmer. The heating of the tissue where the ultrasound is applied can decrease if we vary the angle of incidence. The steeper the angle radiates, the less heating there will be in the tissue [6].

The amount of lysis produced by ultrasound depends on the concentration of the suspended cells. It was shown that the higher the concentration of cells, the less lysis that occurs. This may be due to the high density of cells interfering with the suspended bubbles [28]. This effect could occur because oxygen is dissolved in the breath, thereby increasing CO_2_ and reducing the likelihood of lysis [29]. Ultrasound can stimulate or inhibit cell functions. The authors observed that an exposure of 1 MHz 10 w/cm^2^ in pulses from 20 microseconds to 10 s for 2.5 min can affect cell movement. This causes changes in both the density and volume on the surface of the cells [30]. The maximum intensity at which ablation therapy can be applied is 40 w/cm^2^, since intensities above this level can produce damage to osteocytes and induce thermal necrosis [31]. Low ultrasound intensities, between 12 mW/cm^2^ and 100 mW/cm^2^, can affect bone regeneration. Because of this, ultrasounds are used in the treatment of fractures when applied during the callus-formation process and not in the remodeling phase [32]. The temperature of tissue increases by 1 degree when ultrasound intensities between 20 mW/cm^2^ and 50 mW/cm^2^ are applied, which affects enzymes such as matrix metalloproteinase 1, also known as interstitial collagenase or collagenase I [33]. In spite of the beneficial bone regeneration effects, it should always be considered that ultrasound waves continuously induce thermal effects, and in short but repetitive pulses stimulate cavitation and are associated with cell damage. It is demonstrated that when a tissue with suspended cells is exposed to an ultrasound wave, this wave can produce lysis of those cells [32,33,34,35].

Ultrasound pulses at a low intensity can affect cell-membrane permeability, resulting in increased hydrostatic capillary pressure, thus accelerating fracture healing. Warden et al. [36] observed that treatments of 20 min per day, six days per week for twelve weeks also had no effect on the increased mineralization of femoral or tibial bones in rats [37,38,39,40]. Ultrasound intensities as low as 0.8 W/cm^2^ can produce platelet destruction from vascularization. Erythrocytes are more resistant. However, in the presence of cavitation, hemolysis of ATP has been observed. Moreover, ATP can be released with less intensity in the presence of inertial cavitation. [41] Dalecki et al. [42] observed that 10 microseconds of exposure to repeated pulses of ultrasound at a frequency of 100 Hz for 3 min at 1.2 MHz could cause bleeding from fetal blood vessels. The authors were exposed for 5 min to a 10-microsecond ultrasound at 100 Hz between 0.7 MHz and 3.6 MHz, with areas of bleeding observed above a threshold of radiation pressure 1 MPa. The authors observed that low frequencies produce greater bleeding than high frequencies. Fatemi et al. [43] and Campbell et al. [44] exposed the ears and head of healthy fetuses to ultrasound for 3 min, 10 s to 20 s off, using a scanner equipped with 2 MHz, and compared the results with other fetuses without such exposure or with continuous exposure, thereby observing that fetal movement increased with exposure to the ultrasound. They observed that children who had been exposed to ultrasound more often during pregnancy took longer to speak. 

Kieler et al. [37] and Salvesen [38] observed that the children of mothers who had been exposed to more ultrasound before delivery were more likely to be left-handed. Another Salvesen study [39] reiterates that one will have to live with the uncertainty of whether ultrasound is safe, as there is a slight but real significant difference between ultrasound exposure and being left-handed or right-handed.

Grigor [40] observed that 8 h exposure to 110 dB at 20 kHz in the third-octave band at 20 kHz, 25 kHz and 31.5 kHz did not cause hearing loss in audible frequencies.

People exposed for 15 min to 150 dB at 20 kHz frequency did cause damage to the audible frequencies [45].

Basta et al. [46] observed that in single-layer endothelial cells, ultrasound of variable durations between 1.3 MHz and 2.6 MHz with a mechanical index of 1.5 produced increased intracellular oxidation of endothelial cells in addition to endothelial damage under exposure times greater than 30 s. This damage lasted up to one hour after exposure. After 15 s, it was shown to stagger the DNA and produce leakage of lactate dehydrogenase. The effects on endothelial cells may be increased by pooling ultrasound-exposed extracellular medium with an unexposed extracellular medium or may be eliminated using a cell culture or pretreatment with catalase. Radical formation by inertial cavitation causes intracellular DNA and cell damage until death.

Ultrasounds are used in medicine for diagnostic tests and as treatments for some diseases and injuries. Sound waves produce some mechanical vibrations, known as localized cavitation. These vibrations produce psychochemical changes in the body that cause thermal energy. In the cardiovascular area, this thermal energy generated by ultrasound is used to perform thrombolysis, coronary interventions, drug administration and gene transfer, and to facilitate the recovery of therapeutic injuries [47].

The biological effects due to ultrasound exposure may be due to energy absorption or heating of tissues. Ultrasounds are used for the thermal ablation of tissues in surgery or for therapeutic treatments. These tissues can also be treated with ultrasound by activating the gas in the body with inertial cavitation, causing interactions between bubbles, contrast agents or the pulmonary alveoli. This phenomenon usually occurs at low or medium ultrasound intensities. It also establishes that the Mechanical Index (MI) must be less than 1.9 for such ultrasound to not be harmful [48].

Ultrasound is used in the treatment of cancer tumors and gene transfer via the permeability of cells. For lithotripsy (the removal of kidney stones), waves of 100 kHz to 200 kHz spaced at 1 s intervals are used. The level at which intestinal bleeding occurs is usually higher than the range used for diagnosis. Miller observed that the biological effect of hemolysis under ultrasound usually decreases by increasing the frequency.

Mornstein [49] observed that when ultrasound contrast tests were performed, significant tissue damage was produced, especially damage due to microvascularization. Cosyns et al. [50], Tsutsui et al. [51] and Hayat and Senior [52] examined many patients with contrasting ultrasound and did not detect any clinically significant damage.

It was observed that diagnostic ultrasound testing resulted in increased temperature and cavitation. This increase in temperature and cavitation produced mechanical effects that led to the hydrodynamic breakage of hydrogen bonds, an oscillation of these ions, and chemical effects when free radicals were released. These free radicals interacted with the molecular DNA, causing damage to both the DNA and the chromosomes [53,54].

Chen et al. [55] observed that a 1.3 MHz cardiac ultrasound system applied every four cardiac cycles elevated the tropicamide levels in blood plasma, damaging the myocardium after 30 min of exposure to ultrasound devices with an MI between 1.2 and 1.6.

Hynynen et al. [56] observed damage to the Hemat lymphatic barrier with exposure at a frequency of 1.63 MHz, a pulse length of 100 ms and a repetition of 1 Hz for 20 s with a pressure amplitude between 0.7 MPa and 1 MPa in rabbit brain.

Stanton et al. [57] observed that when using a diagnostic ultrasound with exposures for 15 min at 8 MHz, there was a decrease in the number of cells performing mitosis and an increase in dead cells at 4.5 h after exposure.

Ultrasounds are widely used for medical diagnosis, as well as increasingly for therapeutic purposes (Table 5). Understanding the biological effects of ultrasound is important for clinicians and scientists working in this field, because permanent damage to biological tissues can occur at high levels of exposure [58]. In England alone, over two and a half million obstetric ultrasound scans (about four for every live birth) are performed every year [59]. 

Phacoemulsification is the most common technique in cataract surgery. Conrad-Hengerer et al. [60] compared phacoemulsification surgery with ultrasound to cataract extraction using a femtosecond laser. In this work, a prospective study was carried out in which the number of endothelial cells and the corneal thickness after both types of surgery were quantified. For this purpose, each patient included in the study underwent both techniques (one in each eye), and an intraocular lens was subsequently implanted. For phacoemulsification, pulsed emission ultrasound waves were used, with an adjustment to 60% of their energy. Three months later, an endothelial cell count was done, resulting in a significant loss in the eyes subjected to phacoemulsification by ultrasound, as well as in the corneal thickness. It was concluded that the femtosecond laser did not increase the endothelial damage caused by cataract surgery, while the use of ultrasound did, thus showing that ultrasounds are harmful for eyes with low endothelial cell values before undergoing surgery. Ozil is a phacoemulsification cataract surgery system developed in 2005 in which ultrasonic waves with a frequency of 32 kHz were used continuously or with bursts. This technique achieves an increase in the efficiency of emulsification and a reduction in (or disappearance of) the repulsion between the fragments generated. This results in safer surgery, providing more stable vacuum levels in the anterior chamber and reducing the risk of rupture of the posterior capsule [61]. The phacoemulsification with NeoSoniX system [62] with 120 Hz ultrasonic frequency and offered 2 degrees’ oscillatory movement to the longitudinal displacement produced by conventional ultrasonic energy. The tip of this system moves forward and backward without changing its longitudinal dimension, thereby reducing the heat originating from the intermolecular friction caused by the tip of the phacoemulsifier. It succeeded in significantly reducing the risk of thermal injuries, thus optimizing the level and speed of postoperative rehabilitation. 

### 3.3. Ultrasound in the Health Field and the Workplace Environment

Garaj-Vrhovac and Kopjar [63] observed that the staff in a cardiology unit, working with a color Doppler, experienced greater genotoxic damage than the control group that had not used a Doppler. 

The potential effects of exposure to ultrasound can be differentiated according to the route of transmission, both by contact, mainly manifested in the hands during cleaning and degreasing operations; and by air [2].

The occupational exposures to ultrasound that are transmitted by contact and manifest themselves in the body as functional alterations of the nervous system, headaches, vertigo, fatigue, reflex modifications, vasomotor and peripheral turbulations, can cause heating damage to the skin and even to the bones; or cellular damage, with destruction of the own cells by a cavitation phenomenon. 

**Figure 4:**
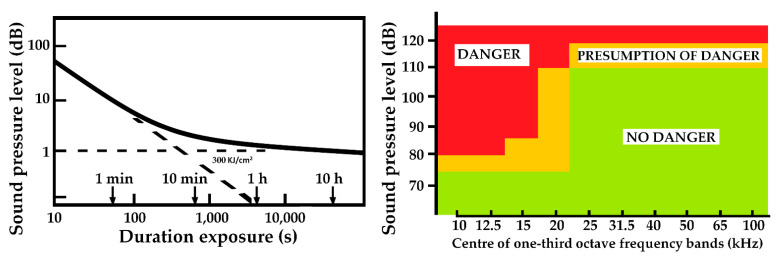
(**Left**) Contact exposure limits proposed by Nyborg in 1978. (**Right**) Recommended maximum limits for exposure to low-frequency airborne ultrasound. Provisional criterion. Status 1985 [2].

Exposure to ultrasound in the air can produce biological effects that manifest themselves in the abnormal development of cells, hematological effects, genetic effects and effects on the nervous system, with symptoms similar to those manifested by exposure through contact. Likewise, the possible displacement of hearing due to the sound components that may accompany ultrasound cannot be ruled out (INSHT, 1985) [2]. Ultrasounds are also used in the industry to make emulsifications, welding and cleaning of utensils [6]. Figure 4 includes this provisional criterion and the contact exposure limits proposed by Nyborg in 1978.

Exposure to the ultrasound of airplanes is mentioned as the cause of symptoms such as nausea, fatigue, dizziness and fullness of the ear [64,65,66]. The question of whether or to what extent airborne ultrasound also causes noise-induced hearing loss is subject to ongoing investigation. Data analysis from 131 measurements at ultrasound-sociated workplaces in Germany performed by the Institute for Occupational Safety and Health (IFA) and by the German Social Accident Insurance Institution for the energy, textile, electrical and media products sectors (BGETEM) show that threshold transgressions occur at a considerable number of workplaces at ultrasonic welding machines of certain frequencies [67].

Kilpatrick [68] listed some decibel ranges that do not produce any observable harm in humans. For instruments and handheld equipment with turbines, the intensity is between 70 dB and 90 dB, with 91 dB for ultrasonic cleaners, 86 dB for ultrasonic scalers, 84 dB for stone-mixing machinery and 74 dB for low-speed handpieces. In dentist tools, it is recommended that the distance between the professional’s eye and the patient’s mouth be 35 cm, since if the professional is closer, he or she will perceive the ultrasound signal emitted by the appliance more highly. Kilpatrick observed that a greater intensity of ultrasound is generated through contact than through air.

Low-frequency ultrasonic technological devices, including washers, welders, drills, soldering tools and galvanizing pots, are the main sources of ultrasonic noise in occupational settings. Ultrasounds are also generated by compressors, pneumatic tools and high-speed machinery such as planers, millers, grinders, circular saws and some textile machinery. Plasma-arc welding, air-acetylene welding, etc. also generate ultrasound [13,69,70,71]. Workers using ultrasonic devices suffer from functional changes such as neurasthenia, cardiac neurosis, hypotension, heart rhythm disturbances (bradycardia) and adrenergic system disturbances [70]. Studies show that exposure to sounds with a frequency of 21 kHz and level of 110 dB for 3 h daily for 10–15 days causes functional changes in the cardiovascular and central nervous systems [72]. Workers exposed to the noise emitted by ultrasound devices suffered from increased neural excitability, irritation, memory problems and difficulties with concentration and learning [64].

In Poland, ultrasonic noise is defined as noise containing high and low ultrasonic frequencies from 10 kHz to 40 kHz. It is estimated that more than 25,000 employees are exposed to this type of noise emitted by ultrasound-technology devices (mainly by ultrasound cleaners) [73].

Grzesik and Pluta [74] performed audiometry on 55 operators of industrial ultrasound devices (Uls) at frequencies of 0.5–20 kHz and compared the results to those of 189 unexposed persons. For workers exposed to US, the authors observed that in addition to presenting threshold elevations in the range of 10–20 kHz, a decreasing number of subjects responded to the stimuli at higher auditory frequencies. 

Smagowska [75] conducted a study with 218 industry employees, 90% of whom were exposed to noise throughout their shifts and stated that most of their work environments had noise sources such as ultrasonic washers, acetyl-oxygen burners, compressed-air valves, pneumatic tools, grinders, metal saws and high-speed cutting machines. Most employees described the noise as buzzing, insistent, high-pitched, squeaking and whistling. Respondents considered the related noise levels as loud, immediate, highly strenuous and exhausting (approx. 55% for each term). The highest number of points on a scale corresponding to noise annoyance was achieved, using descriptors such as horrible, very persistent and intense. 

New measurements indicate that the public is being exposed, without their knowledge, to airborne ultrasound, and that existing guidelines are insufficient for such exposure. Early studies reported changes in hearing thresholds, nausea, headaches, fatigue, migraines and tinnitus, but there is not enough research on human subjects, nor sufficient measurements of the relevant fields, to assess what health risks current occupational and public exposure might produce. In addition, the authors claim that the assumptions underlying audiology and physical measurements at high frequencies need to be questioned; a simple extrapolation from approaches used at lower frequencies does not resolve the current unknowns [76].

Hospital workers with long-term exposure to ultrasound at work may develop dose-dependent neurovascular disorders of the peripheral nervous system in the form of angiodystonic vegetative polyneuritis syndrome of the hands. In some Scandinavian studies, female physiotherapists (exposed to ultrasound and short waves) showed an increased rate of miscarriages and congenital malformations, but no definite conclusions can be drawn based on these results alone. Exposure trends to diagnostic ultrasound equipment over the past two decades show a continuing increase [77].

Maccà et al. [78] performed audiometry on 24 industrial subjects exposed to ultrasound, 113 subjects exposed to industrial noise and 148 unexposed subjects in order to investigate the effects of age, ultrasound and noise on high-frequency hearing thresholds. The subjects exposed to ultrasound had significantly higher hearing thresholds than those not exposed to high frequencies, with the highest ranging from 10 to 14 kHz.

### 3.4. Legislation

France determines the permissible values of ultrasonic noise and recommendations by limiting exposure in the audible range of high frequencies (8–20 kHz) and the low-frequency ultrasonic range (20–50 kHz). In Poland, ultrasonic noise for practical reasons includes both high-frequency audible and low-frequency ultrasonic sound (i.e., 10–40 kHz) [3]. The main sources of ultrasonic noise in the working environment are low-frequency ultrasonic devices such as ultrasonic washers, welding and erosion machines, and manual soldering irons [64].

In Germany, in September 2012, the guideline VDI 3766 was released: “Ultrasound—Workplace—Measurement, Evaluation, Assessment and Reduction” [12]. This guideline describes the specific procedure to measure, evaluate and classify sound exposure from ultrasound in the air. There are no evaluation criteria available to prevent the possible damage caused by airborne ultrasound frequencies to the human ear at frequencies above 8 kHz, as comprehensive and authoritative studies are not available [79].

Poland has standards on the maximum admissible levels (Table 6).

Smagowska and Pawlaczyk-Łuszczyńska [64] concluded in their study that although overexposure to ultrasonic noise was observed among most welders, no significant progress in hearing impairment was observed using Permanent Thresholds Assessment (PTA) after exposure lasting up to 7 years. Since the introduction of exposure limits, few data have shown permanent threshold shifts resulting from occupational exposure to ultrasonic noise. Further studies on the hearing status of workers exposed to ultrasonic noise are needed. The ISO 1999:1990 [81] method for calculating noise-induced permanent threshold shifts might also facilitate reliable predictions of threshold shifts after exposure to ultrasonic noise. 

For occupational safety, measuring airborne ultrasound in situ is vital. However, current measurement techniques apply to measuring audible sound only and do not cover ultrasound for several reasons. The ultrasound fields emitted by today’s industrial appliances are mostly unknown and likely to be complex. Additionally, no weighting for a comparable assessment of exposure to ultrasonic noise has been defined. For example, welding machines, cleaning vessels and cutting machines all vary in size and working frequency, i.e., the frequency that is used to achieve the desired effect. Problematically, such machines usually use high power and thus emit ultrasound at high levels. The limits and guidance levels for ultrasound exposure were ultimately defined in several past publications (e.g., Assessment of noise regarding the risk of hearing damages (VDI 2058-2, 2008) [10,11] and Ultrasound—Workplace—Measurement, assessment, judgement and reduction (VDI 3766, 2012) [12], respectively [82].

The International Electrotechnical Commission (IEC) established that for the ultrasound equipment used in physiotherapy, the maximum temperature threshold of the tissue exposed to ultrasound is 41 °C, always performed on water with a temperature of 25 °C and an intensity of 3 W/cm^2^. Intensities higher than 3 W/cm^2^ will produce an increase in the temperature of the tissue being exposed to ultrasound, thereby producing damage to that tissue. This usually occurs mainly on the surface of the bones. In the CEI EN 61689: 2013 Ultrasonics—Physiotherapy Systems—Field specifications and methods of measurement in the frequency range 0.5 MHz to 5 MHz [83], the maximum intensity that can be applied in physiotherapy treatments is limited. The specified maximum value of 3 W/cm^2^ is a widely established value that accounts for safety conditions and clinical practice. However, for special treatments, lower values may be required depending on the clinical application.

The National Council on Radiation Protection and Measurements of the United States (NCRP) [84] that when the Image Forming (IF) is greater than 0.5 or the thermal index (TI) is greater than 1, the risks produced by the device must be compared to the benefits.

The guidelines for the safe use of diagnostic ultrasound equipment [85] are intended to help manufacturers and operators ensure the prudent use of diagnostic ultrasound. Manufacturers are required to ensure that they have a license obtained from the Licensing Division of Health Canada’s Medical Devices Bureau for the machines that they rent and sell. This update replaces the US parts of Safety Code 23 “Guidelines for the Safe Use of Ultrasound-Part 1: Medical and Paramedical Applications (1989)” [86]. This guide estimates the maximum temperature to which tissue should be exposed, Table 7.

This guidance document “Marketing Clearance of Diagnostic Ultrasound Systems and Transducers” Guidance for Industry and Food and Drug Administration Staff Document issued on 27 June 2019 [87] provides detailed recommendations for manufacturers seeking marketing clearance for diagnostic ultrasound systems and transducers. The manufacturer should indicate that the acoustic output exposure levels were measured, calculated and derated following the most recently released revision of the FDA-recognized consensus standard IEC 62359, along with a declaration of conformity. Alternatively, the measurement procedure should be fully described.

The Table 8 (below) lists the highest known acoustic field emissions for the reamendment’s diagnostic ultrasound devices. The values are derated. Systems that exceed these application-specific acoustic output exposure levels should be evaluated on a case-by-case basis.

Manipulators should employ exposure levels that are as low as reasonably achievable (ALARA) because of the potential for tissue heating. The Thermal Index (TI) and Mechanical Index (MI) should also be considered. It is recommended that the maximum attainable values for the Mechanical Index and the derated spatial peak-time average intensity, ISPTA.3, not exceed 1.9 and 720 mW/cm^2^, respectively.

The intensities and temperature limits are given depending on the type of organ and whether it is fetal. For example, for fetal heart rate monitors, the maximum transducer intensity should be less than 20 mW/cm^2^ two for continuous wave devices [88]. 

There are no significant demonstrated mechanical risk effects from exposure to diagnostic ultrasound. If the MI is exceeded, there is a small risk of pulmonary hemorrhages in fetal or infant tests. 

The methods of measurement are described in the Acoustic Output Measurement Standard for Diagnostic Ultrasound Equipment [26]. The methods of computation are described in the Standard for Real-Time Display of Thermal and Mechanical Acoustic Output Indices on Diagnostic Ultrasound Equipment [27]. 

Diagnostic US exposure that produces an in situ temperature increase of no more than 1.5 °C above normal physiological levels (37 °C) can be used clinically without reservation. If the temperature of the embryo in vivo exceeds 41 °C for 5 min, it should be considered potentially harmful. The time/temperature combination uses the NCR formula (NCRP 1992) t = 4 5^−ΔT^.

There are international regulations for the use of ultrasound at the medical level, Table 9, which have been developed and applied between 2006 and 2010.

Ullisch-Nelken et al. [83] stated that while there has been qualitative and quantitative research on US workplace boundary violations in Germany [94,95] and Poland [96,97], this is the first systematic survey of the distribution of these jobs in Germany. To the knowledge of the authors of this paper, Poland is the only country where such efforts have been made [96,98]. Due to the potential bias of the data set, further research is needed for a real understanding of the distribution of US issuing machinery in workplaces. Transgression of the L_EXAU_, 8 h limit is the best known, which resembles the conventional A-weighted exposure to noise levels present with US. As ultrasound has different properties to audible sound due to its much higher frequency, a thorough investigation of the method applied to ultrasound measurement and its possible influence on workers and spatial parameters on the measurement result is proposed. The research should also consider existing measurement procedures in an international, or at least European, context.

France determines admissible values of ultrasonic noise and recommends limiting noise exposure in the high frequency range (8–20 kHz) and the low frequency range (20–50 kHz) [64].

The current draft Standard prPN-Z_01339:2019 specifies both a method for measuring ultrasonic noise in the work environment and a method for determining equivalent sound pressure levels of ultrasonic noise. The scope of the measurements includes sound pressure levels in the third-octave bands with the center frequencies from 10 kHz to 40 kHz [80]. 

## 4. Discussion 

Many studies show specific values about the behavior of ultrasound waves when applied to different tissues of the human body. Our body is made up of a superposition of tissues whose thickness and composition varies significantly according to sex and age. It would be interesting to carry out studies in which this is considered in order to better control the doses to be applied in therapeutic treatments [6,14,15,16,17,18]. 

The application of ultrasound for ultrasound scans and physiotherapy treatments is carried out at very low doses and its application is controlled, so that the risks of absorption should not be problematic if applied correctly [6,14,15,16,17,18,19,20,21,22,23,24,25,26,27]. Treatments on the human body where cavitation is claimed to occur would have to be specially controlled [6,19,20].

There is a need for the development of improved control procedures or the creation of control protocols that considered parameters such as MI and TI in those applications of US on the human body that do not currently have them [22,23,24,25,26,27].

It is an established fact that the human body absorbs US waves and that the effects produced by this absorption can range from positive to very negative depending on the form of application and the dose [28,29,30,31,32,33,34,35,36,37,38,39,40,41,42,43,44,45,46,99,100,101,102,103]. Nonaudible sound waves are absorbed by our body, and the effects of this absorption in the medium-to-long term are unknown [86]. 

Ultrasound applied in medical treatments may produce unwanted side effects of different levels [47,48,49,50,51,52,53,54,55,56,57,58,59,60,61,62,63] that justify an updated training of all health professionals using them to minimize the risks.

Multiple studies refer to the negative effects on workers who experience ultrasound in their work environments [64,65,66,68,69,70,74,75,76,77,78]. State and international instances have developed increasingly exhaustive studies and reports on the undesirable effects of ultra-sound on human beings [3,12,67,73,81]. It is necessary to combine all this information to define limits that are dependent on frequency and exposure time for the working environment (industry, health, aviation, etc.) and all people in general. 

According to a report by the World Health Organization (WHO) [104], the general population can be exposed to ultrasound from several sources, including consumer sources, exemplified by ultrasonic cleaners, remote control devices, sonar devices, dog control and repulsion devices and distance-measurement devices for cameras, among others, and public sources, exemplified by sources in public areas, such as door openers, burglar alarms, bird and rodent control devices, etc. Currently, many machines that emit ultrasound to scare away animals can be bought on the internet for aesthetic and even therapeutic purposes. There is no reference to these types of ultrasound emitters in public administration documents and it would be interesting to consider any control of their sale and use. 

The energy of ultrasonic waves is absorbed by our body. The depth and form of absorption of this energy is largely unknown depending on the frequency of the acoustic waves, whether they are kHz or MHz. The effects of their absorption are unknown. There is no regulation developed in the European community for its control, either at the occupational, therapeutic or personal level.

## 5. Conclusions

Studies by medical associations and governmental bodies describe the recommended doses and form of application for ultrasound used both physiotherapy and medicine. These documents make no reference to ultrasound-emitting devices used to scare away animals in the home or to cosmetic devices that are sold unchecked on the internet for private use. Consumer protection authorities should consider the medical literature on ultrasound for better control of equipment sold to people for their homes.

Regulations are being developed on exposure times and maximum doses for workers susceptible to ultrasound. There is no mention in these documents that similar doses could be received by people whose homes are near ultrasound-emitting equipment. Both public and private developers should consider possible ultrasound emitters in the vicinity of dwellings for measurement and control.

It is advisable to carry out more studies on the effects of ultrasound on people who receive ultrasound to be able to predict and avoid the negative consequences that these inaudible sounds produce on human beings, since we are increasingly exposed to these acoustic waves whose consequences are ignored by most people.

## Figures and Tables

**Table 1:** Ultrasound applications. NTP 205: Ultrasound: occupational exposure. (INSHT, 1985) [2].

Application	Frequency (kHz)	Intensity Range (W/cm^2^)
Low-frequency underwater signals	16–20	—
Aerosol reactions and agitation	16–20	—
Ultrasonic control devices, door opening	25	—
Welding	16–20	3–32
Industrial cleaning and degreasing	20–25	<6
Plastic welding	20	1000
Metal welding	10–60	10,000
Mechanization	20	variable
Extraction	10	500
Automation	20–300	variable
Thickness measurement	300	—
Experimental biological work	760	—

**Table 2:** The absorption of ultrasound energy produces the following effects [15].

Effects	Mechanism
Vasodilation	Release of tissue stimulants. Reduction in muscle tone.
Muscle relaxation	Elimination of tissue stimulants. Post-excitatory depression orthosympathetic.
Increased membrane permeability	Forcing the tissue fluid through. pH less acidic.
Increased tissue regeneration	Mechanical effect.
Thermal effect	Can block conduction. Nervous tissue possesses special sensitivity to ultrasound.
Decrease in pain	Normalization of muscle tone. Decrease in pH.

**Table 3:** Speed of sound in different substances or organs.

Material	Speed (m/s)
Air	331
Fat	1450/1465
Water (50 °C)	1540
Human soft tissue	1540
Liver	1549
Kidney	1561
Blood	1570/1584
Muscle	1585
Non-fatty tissue	1575
Cortical bone	3635
Amniotic fluid	1535

**Table 4:** Ultrasound equipment used to carry out ultrasound scans groups [22,23,24].

Group	Characteristics
Sectorial	Provide a triangular or fan-shaped image format with a small echo emission start base. Used for cardiac and abdominal explorations since they facilitate a costal approach, and to view deep structures. Usual working frequency between 3.5 and 5 MHz.
Convex	Waves have a curved shape and provide a trapezoid-shaped image format. Used in abdominal and obstetrical to view deep structures. Usual working frequency between 3.5 and 5 MHz.
Linear	Provide a rectangular image format. Used for the study of more superficial structures, such as muscles, tendons, breasts, thyroid, vessels, etc. Usual working frequency between 7.5 and 13 MHz, although some go up to 20 MHz.
Intra-cavity	Linear or convex. Used for intrarectal or intravaginal examinations. Usual working frequency between 5 and 7.5 MHz

**Table 5:** Examples of the use of ultrasound and its frequencies.

Ultrasound Use	Frequencies
Clean jewelry, lenses, watches, instruments	20–40 kHz
Clean teeth (break down bacterial plaque)	1.6 MHz
Lithotripsy	100–200 kHz
Phacoemulsification cataract (torsional Ozil)	32 kHz
Phacoemulsification cataract (NeoSoniX system)	120 Hz

**Table 6:** Maximum admissible Intensities (MAI): Value for Ultrasonic Noise [80].

1/3-Octave Band Frequency, *f* (kHz)	L_f, eq, 8 h, adm_ L_f, eq, wk, adm_ (dB)	L_f, max, adm_ (dB)
10, 12.5, 16	80	100
20	90	110
25	105	125
31.5, 40	110	130

L_f, eq, 8 h, adm_ is the equivalent continuous sound pressure levels in the 1/3-octave bands, normalized to a nominal 8 h daily work. L_f, eq, wk, adm_ is the equivalent continuous sound pressure levels in the 1/3-octave bands normalized to a 40 h workweek. L_f, max, adm_ is the maximum sound pressure levels in the 1/3-octave bands.

**Table 7:** Temperature level limits [86].

Temperature Increase	Maximum Time Exposure
39 °C (2 degrees above normal)	60 min
40 °C (3 degrees above normal)	15 min
41 °C (4 degrees above normal)	4 min
42 °C (5 degrees above normal)	1 min
43 °C (6 degrees above normal)	0.25 min

**Table 8:** Maximum admissible Intensity (MAI): Value for Ultrasonic Noise [88].

Use	I_SPTA.3_ (mW/cm^2^)	I_SPTA.3_ (W/cm^2^)	MI
Peripheral Vessel	720	190	1.9
Cardiac	430	190	1.9
Fetal Imaging and Other	94	190	1.9
Ophthalmic	17	28	0.23

**Table 9:** International regulations related to the medical use of ultrasound.

Name of Regulation	Reference
IEC60601-2-37 Edition 2. 2007	[89]
IEC61157 Edition 2.0. 2007	[90]
IEC61161 Edition 2.0. 2006	[91]
IEC62127-1 Edition 1.0. 2007	[92]
IEC62359 Edition 2.0. 2010.	[93]
